# Supplementary material for: Antitumor Effect of Regorafenib on MicroRNA Expression in Hepatocellular Carcinoma Cell Lines
Source: Int J Mol Sci. 2022 Jan 31;23(3):1667. doi: 10.3390/ijms23031667 (PMC8835935; doi:10.3390/ijms23031667)
Supplement: Supplementary file 1 [file ijms-23-01667-s001.zip › ijms-1546379-supplementary.pdf]

International Journal of *Molecular Sciences*

Supplementary Materials

Antitumor Effect of Regorafenib on MicroRNA Expression in Hepatocellular Carcinoma Cell Lines

Kei Takuma [1], Shintaro Fujihara [1], Koji Fujita [1], Hisakazu Iwama [2], Mai Nakahara [1], Kyoko Oura [1], Tomoko Tadokoro [1], Shima Mimura [1], Joji Tani [1], Tingting Shi [1], Asahiro Morishita [1], Hideki Kobara [1], Takashi Himoto [1] and Tsutomu Masaki [1,*]

[1] Department of Gastroenterology and Neurology, Faculty of Medicine, Graduate School of Medicine, Kagawa University, 1750-1 Ikenobe, Miki-cho, Kita-gun, Kagawa 761-0793, Japan; takuma.kei@kagawa-u.ac.jp (K.T.); fujihara.shintaro@kagawa-u.ac.jp (S.F.); fujita.koji@kagawa-u.ac.jp (K.F.); nakahara.mai@kagawa-u.ac.jp (M.N.); oura.kyoko@kagawa-u.ac.jp (K.O.); tadokoro.tomoko@kagawa-u.ac.jp (T.T.); mimura.shima@kagawa-u.ac.jp (S.M.); tani.joji@kagawa-u.ac.jp (J.T.); shi.tingting@kagawa-u.ac.jp (T.S.); morishita.asahiro@kagawa-u.ac.jp (A.M.); kobara.hideki@kagawa-u.ac.jp (H.K.); himoto@chs.pref.kagawa.jp (T.H.)

[2] Life Science Research Center, Kagawa University, 1750-1 Ikenobe, Miki-cho, Kita-gun, Kagawa 761-0793, Japan; iwama.hisakazu@med.kagawa-u.ac.jp

* Correspondence: tmasaki@med.kagawa-u.ac.jp; Tel.: +81-87-891-2156

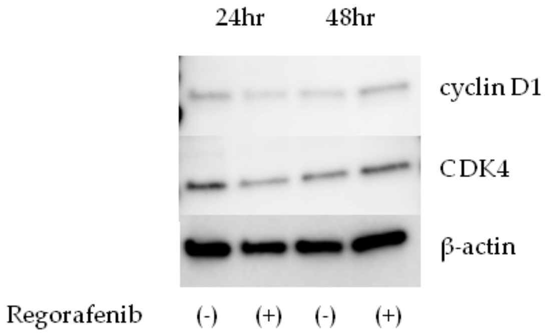

Figure S1. Western blot showing the expression of cyclin D1 and CDK4 in PLC/PRF/5 cells at 24 h and 48 h after the addition of 5 μM regorafenib.
